# Efficient Lane Boundary Detection with Spatial-Temporal Knowledge Filtering

**DOI:** 10.3390/s16081276

**Published:** 2016-08-12

**Authors:** Zhixiong Nan, Ping Wei, Linhai Xu, Nanning Zheng

**Affiliations:** Institute of Artificial Intelligence and Robotics, Xi’an Jiaotong University, Shaanxi 710049, China; nanzhixiong@stu.xjtu.edu.cn (Z.N.); xlh@xjtu.edu.cn (L.X.); nnzheng@xjtu.edu.cn (N.Z.)

**Keywords:** lane detection, spatial-temporal knowledge, crossing point filter, structure triangle filter

## Abstract

Lane boundary detection technology has progressed rapidly over the past few decades. However, many challenges that often lead to lane detection unavailability remain to be solved. In this paper, we propose a spatial-temporal knowledge filtering model to detect lane boundaries in videos. To address the challenges of structure variation, large noise and complex illumination, this model incorporates prior spatial-temporal knowledge with lane appearance features to jointly identify lane boundaries. The model first extracts line segments in video frames. Two novel filters—the Crossing Point Filter (CPF) and the Structure Triangle Filter (STF)—are proposed to filter out the noisy line segments. The two filters introduce spatial structure constraints and temporal location constraints into lane detection, which represent the spatial-temporal knowledge about lanes. A straight line or curve model determined by a state machine is used to fit the line segments to finally output the lane boundaries. We collected a challenging realistic traffic scene dataset. The experimental results on this dataset and other standard dataset demonstrate the strength of our method. The proposed method has been successfully applied to our autonomous experimental vehicle.

## 1. Introduction

Lane boundary detection has been extensively studied over the past few decades for its significance in autonomous guided vehicles and advanced driver assistance systems. Despite the remarkable progress achieved, many challenges remain to be addressed. The first challenge is the drastic change of lane structures. For example, a lane may suddenly become wide or narrow. In addition, lane boundary detection is often disturbed by too ‘sufficient’ visual cues in traffic scenes, such as shadows, vehicles, and traffic signs on roads, or contrarily, too weak cues, such as worn lane markings. These challenges often make conventional methods inapplicable or even result in misleading outcomes.

One of the major reasons is that the conventional methods highlight the effect of lane appearance features, but overlook the effect of prior spatial-temporal knowledge. Lanes are knowledge-dominated visual entities. Lane appearances are relatively simple, usually parallel lines. They have no sophisticated structures, textures and features. When the appearance is interfered with by noise, humans often resort to their prior knowledge to identify lanes. For example, in urban scenes where the frontal and passing vehicles occlude the lanes, humans can filter out pseudo lines and estimate the real lane boundaries according to their knowledge of lane width constraints and the lane boundaries at the previous time.

**Figure 1:**
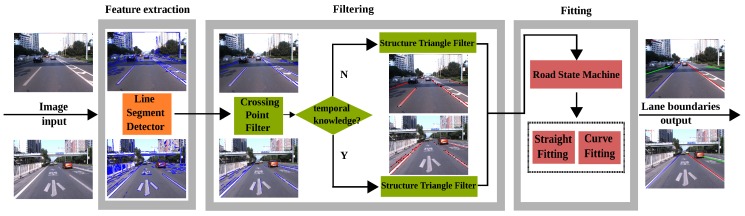
Overview of our method.

In this paper, we propose a spatial-temporal knowledge filtering method to detect lane boundaries in videos. The general framework is shown in Figure 1. This model unifies the feature-based detection and knowledge-guided filtering into one framework. With a video frame, the model first extracts line segment features with the Line Segment Detector (LSD) [1]. This approach differs from traditional edge detection and can obtain robust and accurate line segments in various traffic scenes. A Crossing Point Filter (CPF) and a Structure Triangle Filter (STF) are proposed to filter out noisy line segments. These two filters characterize the spatial structure constraints and temporal location constraints, which represent the prior spatial-temporal knowledge about the lanes. A straight line or curve model that is determined by a state machine is used to fit the line segments and finally to produce the lane boundaries.

The proposed method was tested on a large-scale dataset. This dataset was collected in natural traffic environments under various weather conditions, illumination settings and scenes. The experimental results demonstrate that the method can detect lane boundaries in various challenging scenes with high performance. Moreover, the proposed algorithm has been successfully applied to our autonomous experimental vehicle.

Compared to previous work, this paper makes four major contributions.

1. It develops a framework that incorporates prior spatial-temporal knowledge and appearance features to detect lane boundaries in videos.
2. It proposes two knowledge-based filters to filter out noisy line segments.
3. It builds a large-scale dataset of traffic scene videos. The proposed method was tested on this dataset and achieved impressive performance.
4. The algorithm has been successfully applied to an autonomous experimental vehicle.

## 2. Related Work

In this section, we briefly review related literature from the following major streams: feature extraction, feature refinement, lane fitting and lane tracking.

### 2.1. Feature Extraction

Edges are among the most widely-used features in lane representation and detection [2,3,4,5,6,7,8,9,10,11,12,13,14]. Canny edges [15] are composed of pixels with strong gradient magnitudes. The steerable Gaussian filter [5,16,17,18,19,20,21] extracts edge features by utilizing the gradient orientation information. However, the thresholds to determine edges in these methods are manually set to be constant, which causes the method to be inapplicable to dynamically-changing traffic scenes. Color is another widely-used feature in lane detection [22,23]. However, it is sensitive to illumination changes.

Machine learning methods were recently introduced to feature extraction to overcome those drawbacks [24,25,26,27,28]. The method in [24] trained an artificial neural network classifier to obtain the potential lane-boundary pixels. Such features extracted by an off-line-trained classifier are closely related to the varieties and scales of the training samples. Multiple types of features were fused to overcome the drawbacks of unary features in a previous study [29]. In our work, the LSD algorithm [1] is used to extract lane segment features. This approach can accurately extract line segments in various traffic scenes without manually setting the thresholds.

### 2.2. Feature Refinement

To refine the extracted features, classical image processing algorithms, such as the threshold segment [11,12,13,30,31,32] and the Gaussian filter [4,24,30,33,34,35], are usually employed. These methods need to set the thresholds manually and do not take advantage of the information of road geometry structures.

Filtering methods based on geometry constraints are also explored to refine line features [5,9,10,23,36,37]. For example, the IPM-based methods [3,9,18,20,25,27,30,31,32,33,35,36,38,39,40,41,42] eliminate noise by searching for horizontal intensity bumps in bird’s eye-view images based on the assumptions of parallel lane boundaries and flat roads. However, if roads are not flat, with those methods, lane boundaries will be mapped as nonparallel lines on the bird’s eye-view images, thereby leading to false detection. Additionally, horizontal bumps are difficult to detect in traffic scenes with weak visual cues, such as worn-out lane boundaries and complex illuminations. Moreover, the IPM-based methods require calibration parameters, which causes inevitable systematic error and repeated calibrations if the camera is moved.

Aside from the flat roads and parallel lane boundaries, other geometrical structure constraints are used for feature refinement. Global lane shape information is utilized to iteratively refine feature maps in [5]. The method in [9] utilizes driving direction to remove useless lines. Vanishing points are utilized in both [5,9] to improve the filtering performance. Shape and size information is used to determine whether a region belongs to a lane boundary in [23]. However, the spatial-temporal constraints have not been extensively analyzed.

Searching strategies are also explored to refine feature maps. The model in [43] searches for useful features by employing a scanning strategy from the middle pixel of each row to both sides. An edge-guided searching strategy is proposed in [8]. Kang and Jung [44] detected real lane boundaries using a dynamic programming search method. Expensive sensors, such as GPS, IMU and LIDAR, are utilized to provide assistant information [25,45]. However, these strategies often lack general applicability.

In this paper, we propose two generally applicable filters, namely CPF and STF. They characterize geometrical structure and the temporal location constraint, respectively.

### 2.3. Lane Fitting

Many straight and curve fitting methods have been developed. Hough transform is frequently used for straight line fitting [6,7,8,10,11,25,30,31,35,37,38,43,46,47]. The parabola and hyperbola are classical curve models that are adopted by [5,7,36,38,48,49,50,51,52]. The major limitation of the quadratic curve is the lack of flexibility to model the arbitrary shape of lane boundaries. Therefore, other curves, such as Catmull–Rom [2,34,46,53], B-spline [6,35], Bezier [33] and the cubic curve [4,18,30], are also widely used. Generally, when fitting a lane, many candidates are generated by RANSAC [8,9,18,20,24,25,27,31,32,33,35,38,39,54], and the candidate with the maximum likelihood is chosen.

In this paper, we design a state machine to estimate if a lane is straight or curved. Then, the straight line or curve fitting model is used to fit lanes.

### 2.4. Lane Tracking

Tracking technology is used to improve the computation efficiency and detection performance by utilizing the information of temporal coherence. Among tracking methods, the Kalman filter [43,46,54] and the particle filter [24,28,55] are the most widely used. The model in [55] defines the particle as a vector to represent the control points of lane boundaries. However, such methods often assume that the changes of lane boundary positions between two consecutive frames are small, which may be inapplicable when a vehicle turns at a crossroad or changes lanes. The road paint, heavy traffic and worn lanes also bring challenges to these methods.

## 3. Feature Extraction

We extract line segments in video frames as lane boundary features with the Line Segment Detector (LSD) proposed in [1]. LSD is an efficient and accurate line segment extractor, which does not require manually-set parameters.

**Figure 2:**
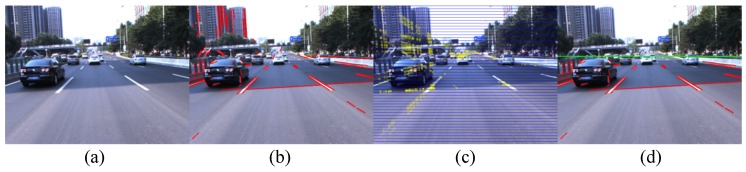
Feature extraction. (**a**) Original image; (**b**) Line Segment Detector (LSD) result; (**c**) Horizontal bands in the image; the yellow points represent the crossing points of line segments; (**d**) Line segments above the vanishing line are eliminated.

The principle process of the LSD extraction is described as follows [1]. An RGB image is first converted to a gray image, which is then partitioned into many line support regions. Each region is composed of a group of connected pixels that share the same gradient angle. The line segment that best approximates each line support region is identified. Finally, all of the detected line segments are validated. Figure 2 shows some procedures of the feature extraction.

To convert an RGB image into a gray image, the gray intensity I(x) at a pixel *x* is represented as a weighted average of the RGB values R(x), G(x) and B(x), i.e., [11],

(1) $$\begin{array}{c} I\left(x\right)=\omega_{1}R(x)+\omega_{2}G\left(x\right)+\omega_{3}B\left(x\right) \end{array}$$

The study [48] has demonstrated that the red and green channels exhibit good contrast properties for white and yellow lane markings. Since most lane markings on real roads are white and yellow, we set $\omega_{1}=0.5,\omega_{2}=0.5$ and $\omega_{3}=0$ in Equation (1) to enhance the contrast of lane markings to surroundings.

The extracted line segments above lanes’ vanishing line are the noise for lane boundary detection. To remove those noisy line segments, we should localize the lanes’ vanishing line. We develop a vanishing line detection method that does not require camera calibration. In our method, we assume that the rotation angle of the camera with respect to the horizontal axis is zero.

To localize the vanishing line, the crossing points of all of the line segments are first computed. Then, the image plane is uniformly divided into horizontal bands, as illustrated in Figure 2c. Each horizontal band is assigned a score. The score of the *i*-th band is,

(2) $$\begin{array}{c} P_{i}=\frac{n_{i}}{\sum_{i=1}^{N}n_{i}} \end{array}$$

where $n_{i}$ is the number of crossing points in the *i*-th band and *N* is the number of bands. In this work, the height of each band is set as 10 pixels.

**Figure 3:**
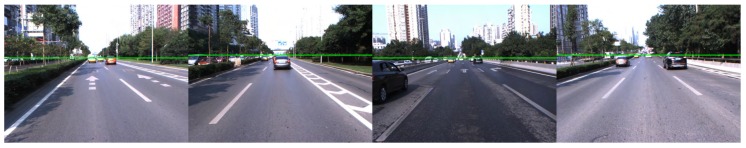
Examples of vanishing line detection.

The horizontal symmetry axis of the band with the highest score is considered as the vanishing line. The line segments above the vanishing line are eliminated, and the remaining segments serve as lane boundary features for subsequent processing, as shown in Figure 2d. Some vanishing line detection examples are shown in Figure 3.

## 4. Filtering

Due to the noise and complex traffic scenes, not all line segment features extracted by LSD are from lane boundaries. The line segment features possibly on the lane boundaries should be kept while the features in other areas should be eliminated. This processing is realized by filtering. In this section, we present two knowledge-based filters that are used to filter out noisy line segments. The filtering reflects and characterizes two types of knowledge, namely spatial geometry constraints and temporal location consistency.

### 4.1. Crossing Point Filter

#### 4.1.1. Definition

According to the camera projection, lane boundaries in a 2D image that are parallel in the 3D world will intersect at the same vanishing point [23]. The general idea of CPF is to filter out those line segments not passing the vanishing point.

**Figure 4:**
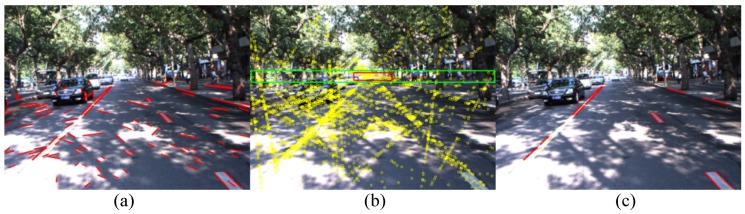
Illustration of the Crossing Point Filter (CPF). (**a**) Original feature map; (**b**) The vanishing box (red box) and the restrictive region (green box); the yellow points are the crossing points of all line segments; (**c**) The CPF result.

However, a single point is prone to be interfered with by noise and difficult to estimate accurately. Inspired by previous studies that use vanishing points to detect lanes [5,9], we use a bounding box near the vanishing point to refine the line segments. We call this bounding box the vanishing box, as the red box shown in Figure 4b. A line segment is filtered out if all of the crossing points of this segment with other segments are outside the vanishing box. Figure 4c shows that many noisy line segments are filtered out.

The vanishing box in the *n*-th frame is define as:

(3) $$\begin{array}{c} b_{n}=\left\{x_{n},y_{n},w_{n},h_{n},s_{n}\right\} \end{array}$$

where $\left(x_{n},y_{n}\right)$, $w_{n}$, $h_{n}$ and $s_{n}$ are the top-left point, the width, the height and the score of $b_{n}$, respectively.

#### 4.1.2. Vanishing Box Search

Since the vanishing box is close to the vanishing line, we search for the vanishing box in a restrictive region *R* centered on the vanishing line, as the green box shown in Figure 4b. The restrictive region *R* is defined as:

(4) $$\begin{array}{c} R=\{r_{x},r_{y},r_{w},r_{h}\} \end{array}$$

where $r_{w}$ is the width of *R* and set as the width of the image. $r_{h}$ is the height of *R* and set as $r_{h}=60$ in our work. $r_{x}=0$ and $r_{y}=v_{0}-0.5r_{h}$ are the top-left point of *R*, where $v_{0}$ is the vertical position of the vanishing line in the image.

Within *R*, we search for the vanishing box $b_{n}$ at the positions by a spacing step d=5 in the horizontal and vertical directions. The candidate box at the local coordinate (i,j) relative to the top-left point of *R* is $b_{n}^{ij}=\left\{x_{n}^{ij},y_{n}^{ij},w_{n}^{ij},h_{n}^{ij},s_{n}^{ij}\right\}$. In the experiment, we set the width and height as $w_{n}^{ij}=W/4$, $h_{n}^{ij}=30$, where *W* is the width of the image.

The position $\left(x_{n}^{ij},y_{n}^{ij}\right)$ in the image is:

(5) $$\begin{array}{c} x_{n}^{ij}=r_{x}+i\cdot d,\;\;\;\;\;y_{n}^{ij}=r_{y}+j\cdot d \end{array}$$

The score $s_{n}^{ij}$ is defined as:

(6) $$\begin{array}{c} s_{n}^{ij}=\;\frac{n_{ij}}{n} \end{array}$$

where $n_{ij}$ is the number of crossing points inside $b_{n}^{ij}$ and *n* is the total number of crossing points inside *R*.

Among all of the candidate boxes, the one with the highest score is identified as the vanishing box $b_{n}$.

### 4.2. Structure Triangle Filter

**Figure 5:**
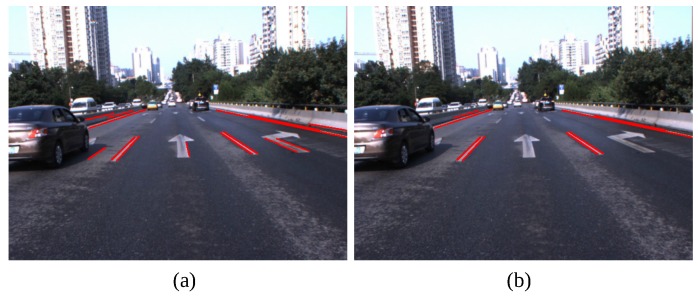
Filtering results. (**a**) Result of CPF; (**b**) Result of Structure Triangle Filter (STF).

CPF cannot filter out the noisy line segments that are parallel to the lanes. For example, in Figure 5a, the noisy line segments on the arrow traffic signs still remain after applying CPF. We present a structure triangle filter (STF) to further remove those noisy line segments that are parallel with the lane boundaries.

#### 4.2.1. Definition

**Figure 6:**
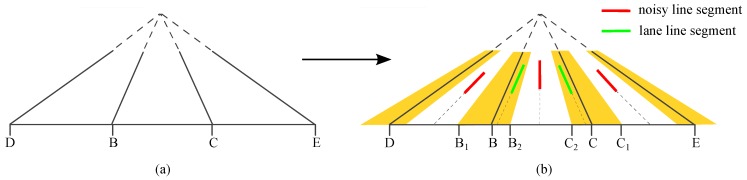
Illustration of the structure triangular filter. (**a**) The structure triangle; (**b**) Line segments filtering with the structure triangle; the yellow regions indicate the lane tolerance regions.

We assume that in the real 3D world, the left and right neighboring lanes have the same width as the ego lane. Based on this assumption, the ego lane and the neighboring lanes form a triangular structure with the bottom line of the image, as shown in Figure 6a. The intersection points of the lanes and the image bottom line, as B,C,D,E in Figure 6a, meet BD=CE=BC.

The line segments in a small neighborhood around a lane are likely to contribute to estimating the lane. We call this neighborhood the tolerance region, as the yellow regions in Figure 6b. The tolerance region can be approximately defined with a range in the image’s bottom line. If a line segment is in a lane’s tolerance region, the segment’s intersection point with the image bottom will be in a small neighborhood of the lane’s intersection point with the image bottom; otherwise, the intersection point will be outside this neighborhood. The similar equal-width lane assumption and constraint were also utilized in previous work [39].

As shown in Figure 6b, the green segments are in the tolerance region, while the red segments are outside; $B_{1}B_{2}$ and $C_{1}C_{2}$ are the small neighborhoods. In the experiment, we empirically set $BB_{1}=2BB_{2}$ and $BB_{1}=BC/8$.

#### 4.2.2. Estimation

To filter out noisy line segments with STF, we should estimate the points *B*, *C*, *D* and *E* in each video frame. To estimate *B*, we identify the line segments from CPF with negative slopes (in the image coordinate system). Among all of the intersection points of these segments with the image’s bottom line, the point with the maximum horizontal coordinate is approximately taken as *B*. *C* is estimated in a similar way, but using the line segments with positive slopes and selecting the point with the minimum horizontal coordinate. With *B* and *C*, *D* and *E* are computed with the constraint BD=CE=BC.

It should be noted that the estimated *B*, *C*, *D* and *E* are not the intersection points of real lane boundaries with the image’s bottom line. After filtering out noisy line segments with STF, the remaining line segments are used to estimate real lane boundaries, which will be detailed in Section 5.

#### 4.2.3. Temporal Knowledge Transition

The STF filtering is based on reasonable estimation of *B* and *C*. Incorrect estimation may lead to misleading results for the subsequent processing. Therefore, the incorrect estimation should be identified. If this occurs, the STF from the previous frames will be applied, which reflects the transition of temporal knowledge about lanes.

In the current frame, let $L_{BC}$ be the length of the estimated BC and $L_{a}$ be a prior constant. If $0.7L_{a}\leq L_{BC}\leq 1.6L_{a}$, the estimated *B* and *C* are taken to be applicable; otherwise, they are inapplicable. $L_{a}$ is the average value of all $L_{BC}$ in the previous frames where *B* and *C* are identified to be applicable. This is computed with Algorithm 1, where $L_{a}$ is empirically initialized. The two empirical values 0.7 and 1.6 slack the range of $L_{BC}$ and make the method more robust to lane drift.

**Algorithm 1:** Computing $L_{a}$

 1:Initialize Q=0, $L_{sum}=0$, $L_{a}$ 2:**while** (capture frame) **do**
 3: **if** $L_{BC}$ applicable **then** 4:  $L_{sum}+=L_{BC}$ 5: **else** 6:  $L_{sum}+=L_{a}$ 7: **end if** 8: Q=Q+1 9: $L_{a}=L_{sum}/Q$10:**end while**


## 5. Fitting

The line segments output from CPF and STF are further used for fitting lane boundaries. Since straight and curved lanes both occur in traffic scenes, we should adopt different fitting models. We present a road state machine to determine if a lane is straight or curved. Then, the corresponding line or curve model is selected to fit lane boundaries.

### 5.1. Road State Machine

**Figure 7:**
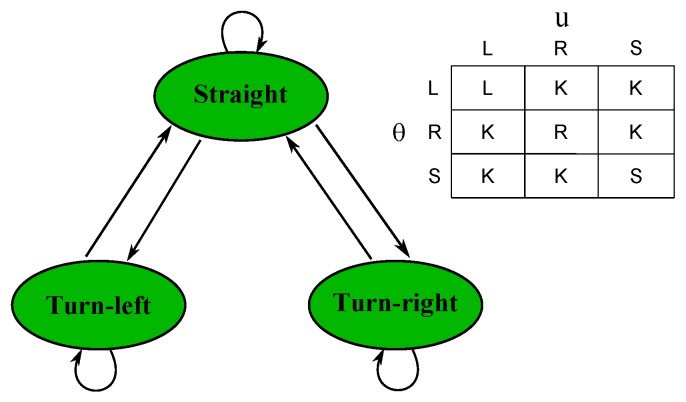
Road state machine. L, R, S and K denote ’left’, ’right’, ’straight’ and ’keep’, respectively.

The state machine includes three states: turn-left road, turn-right road and straight road, as shown in Figure 7. We assume that the state cannot directly transfer between ‘turn-left’ and ‘turn-right’ in two consecutive frames. The road state is jointly decided by two types of measures. Only if the two measures indicate the same state, the state of the current road is assigned the indicated state. When the two measures indicate different states, the current road state is assigned the state in the last frame.

As discussed in Section 4.2, line segments in tolerance regions contribute to estimating lane boundaries. The first measure is the difference between the inclination angles of the line segments respectively in the left boundary’s tolerance region and the right boundary’s tolerance region. It is defined as:

(7) $$\begin{array}{c} \theta =\arctan\left(\frac{1}{n_{1}}\sum_{k=1}^{n_{1}}|\frac{j_{1}^{k}-j_{2}^{k}}{i_{1}^{k}-i_{2}^{k}}|\right)-\arctan\left(\frac{1}{n_{2}}\sum_{k=1}^{n_{2}}|\frac{j_{3}^{k}-j_{4}^{k}}{i_{3}^{k}-i_{4}^{k}}|\right) \end{array}$$

$\left(i_{1}^{k},j_{1}^{k}\right)$ and $\left(i_{2}^{k},j_{2}^{k}\right)$ are the endpoints of the *k*-th line segment in the left boundary’s tolerance region. $\left(i_{3}^{k},j_{3}^{k}\right)$ and $\left(i_{4}^{k},j_{4}^{k}\right)$ are the endpoints of the *k*-th line segment in the right boundary’s tolerance region. $n_{1}$ and $n_{2}$ are the segment’s numbers in the two regions, respectively.

For *θ*, we introduced a positive threshold Δ. If θ>Δ, which means that the inclination angle of the left boundary is larger than the angle of the right boundary, the road state will be ‘turn-left’. If -Δ≤θ≤Δ, which means that the inclination angles of the two boundaries are close, the road state will be ‘straight’. If θ<-Δ, which means that the inclination angle of the left lane is smaller than the angle of the right lane, the road state will be ‘turn-right’. In our experiment, Δ is set as π/9 empirically.

The second measure is the horizontal coordinate *u* of the lane’s vanishing point. Let *W* be the image width and $\Delta_{1}$ be a positive threshold. If $u<0.5W-\Delta_{1}$, which means the vanishing point is in the left side of the image, the road state will be ‘turn-left’. Similarly, $0.5W-\Delta_{1}\leq u\leq 0.5W+\Delta_{1}$ and $u>0.5W+\Delta_{1}$ indicate the ‘straight’ and ‘turn-right’ states, respectively. In our experiment, $\Delta_{1}$ is set as W/16 empirically.

With the indications of *θ* and *u*, we can decide if a road is straight or curved. Figure 7 shows the road state decision table.

### 5.2. Lane Fitting

#### 5.2.1. Straight Lane

For a straight boundary, we use a line to fit the line segments in a tolerance region (as a yellow region in Figure 6b). The line slope *a* and a point $\left(x_{0},y_{0}\right)$ on the line are defined as:

(8) $$\begin{array}{c} a=\frac{1}{N}\sum_{i=1}^{N}\frac{y_{1}^{i}-y_{2}^{i}}{x_{1}^{i}-x_{2}^{i}},\;\;\;x_{0}=\frac{1}{N}\sum_{i=1}^{N}\frac{x_{1}^{i}+x_{2}^{i}}{2},\;\;\;y_{0}=\frac{1}{N}\sum_{i=1}^{N}\frac{y_{1}^{i}+y_{2}^{i}}{2} \end{array}$$

where $\left(x_{1}^{i},y_{1}^{i}\right)$ and $\left(x_{2}^{i},y_{2}^{i}\right)$ are the end points of line segments, while *N* is the line segment number.

#### 5.2.2. Curved Lane

We use the Catmull–Rom model for curved lanes [2,53]. Since curves are prone to be interfered with by noise, with the line segments in each frame, we generate all candidate Catmull–Rom curves for the left and right boundaries of a lane. The curve pair of the left and right boundaries that is most similar to the curve pair of the last frame is identified as the final results.

The similarity of the lane boundary pairs in two consecutive frames is defined as:

(9) $$\begin{array}{c} f=\frac{1}{m}\sum_{i=1}^{m}\left(1-w_{i}*\frac{|L_{n+1}^{i}-L_{n}^{i}|}{L_{n+1}^{i}}\right) \end{array}$$

**Figure 8:**
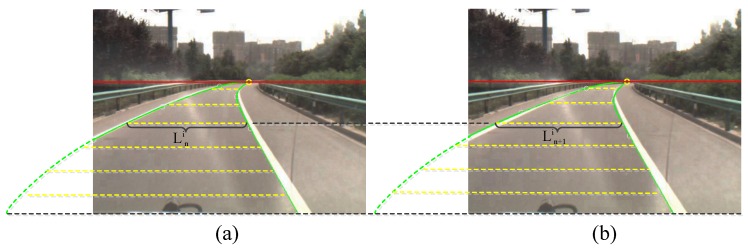
Shape similarity of lane boundaries in two consecutive frames. (**a**) Lane boundary detection in the *n*-th frame; (**b**) Lane boundary detection in the (n+1)-th frame.

We use Figure 8 to illustrate Equation (9). The green curves in Figure 8a are the lane boundary results in the *n*-th frame, and the curves in Figure 8b are the results in the (n+1)-th frame. The red line is the vanishing line, and the yellow dotted lines represent the lane width in different rows. *m* is the number of rows between the vanishing line and the bottom line. $L_{n}^{i}$ is the width of the lane in the *i*-th row of the *n*-th frame, and $L_{n+1}^{i}$ is the width in the same row of the (n+1)-th frame. $w_{i}$ is a penalty factor, which is defined as $w_{i}=L_{n}^{i}/L_{n}^{m}$, where $L_{n}^{m}$ is the width of the lane on the bottom line of the image in the *n*-th frame.

**Figure 9:**
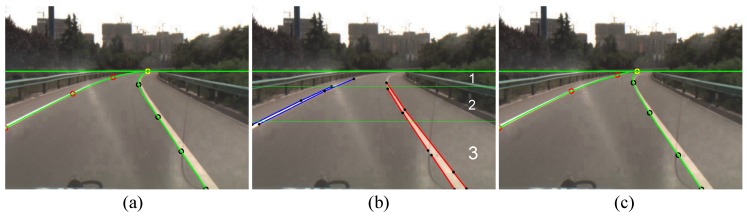
Curve lane boundary fitting. (**a**) Lane boundaries and control points in the *n*-th frame; (**b**) Computing the control points in the (n+1)-th frame; (**c**) Lane boundaries and control points in the (n+1)-th frame.

We define a Catmull–Rom curve with five control points $p_{i}=\left(x_{i},y_{i}\right),i=1,2,3,4,5$. The vertical coordinate of $p_{1}$ is set as the vertical coordinate of the vanishing line, and its horizontal coordinate is estimated by the curve vanishing point estimation method [34]. $p_{2}$, $p_{3}$ and $p_{4}$ are distinctly located at three horizontal regions with unequal heights, respectively, as shown in Figure 9b. In their distinct regions, $p_{2}$, $p_{3}$ and $p_{4}$ are set as the endpoints of the line segments. By assigning all of the endpoint combinations to $p_{2}$, $p_{3}$ and $p_{4}$, all of the candidate curves are generated. $p_{5}$ is set as the crossing point of the image bottom line and the line segment containing $p_{4}$. For curve fitting, two assistant points, $p_{0}=\left(x_{0},y_{0}\right)$ and $p_{6}=\left(x_{6},y_{6}\right)$, are empirically defined as $x_{0}=2x_{1}-x_{2}$, $y_{0}=y_{2}$, $x_{6}=x_{5}$, $y_{6}=2y_{5}-y_{4}$.

## 6. Experiments

### 6.1. Dataset and Setting

We collected a large-scale realistic traffic scene dataset: the XJTU-IAIR traffic scene dataset. It includes about 103,176 video frames. The dataset covers: (1) various kinds of lanes, such as dashed lanes, curve lanes, worn-out lanes and occluded lanes; (2) diverse road structures, such as wide roads, narrow roads, merging roads, tunnel roads, on-ramp roads, off-ramp roads, irregular shape roads and roads without lane boundaries; (3) various disturbances, such as shadows, road paint, vehicles and road water; (4) complex illumination conditions, such as day, night, dazzling, dark and illumination change; and (5) different behaviors of ego vehicles, such as changing lanes and crossroad turning. In addition to lane detection, this dataset can also be used in traffic scene understanding, vehicle tracking, object detection, etc. For lane detection, we tested our method on parts of videos in this dataset.

The dataset is organized as follows. We first categorize the videos into highway and urban videos. In each part, the videos are classified into general ones and particular ones. The general videos are longer and include different kinds of traffic scenes, while the particular videos are shorter and focus on certain traffic conditions, such as illumination, curve, night, changing lanes, etc. Table 1 summarizes the statistics of our dataset.

We also tested our algorithm on Aly’s dataset [33], which is a well-known and well-organized dataset for testing lane detection. It includes four video sequences. It was captured in real traffic scenes with various shadows and street surroundings.

Experiments 1, 2 and 3 were performed on a PC platform equipped with an Intel i7 CPU with a quad-core of 2.8 GHZ at the frame resolution of 640×480. Experiment 4 was conducted on different platforms with lower computing ability.

### 6.2. Evaluation Criterion

We use the criteria of precision and recall to evaluate the performance of the methods, which are defined as:

(10) $$\begin{array}{c} \begin{array}{c} Precision=\frac{TP}{TP+FP}\;\;,\;Recall=\frac{TP}{TP+FN} \end{array} \end{array}$$

where TP is the total number of true positives, FP is the total number of false positives and FN is the total number of false negatives.

**Figure 10:**
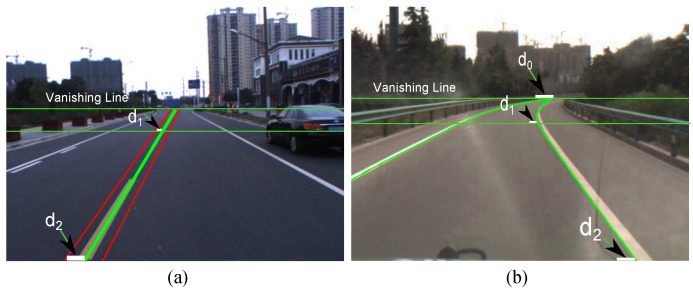
Evaluation criterion. (**a**) Straight lanes; (**b**) Curved lanes.

The detected boundary is taken as a true positive if the horizontal distances between the detected boundary and the ground truth at several different positions are all less than the predefined thresholds. For straight lanes, this rule is checked at two positions: the bottom line and the line that divides the distance between the bottom line and the vanishing line as 5:1, as shown in Figure 10a. For curved lanes, an additional check is made at the vanishing line, as shown in Figure 10b. In the experiments, we manually count the number of true positives, false positives and false negatives with the same standard. To have a fair comparison with other methods, we compute the performance with the results of ego lanes. In the following experiments, we use GT to denote the total number of ground truth and FPS to denote frames per second.

### 6.3. Experiment 1: XJTU-IAIR Traffic Scene Dataset

We evaluate our method on parts of videos in our XJTU-IAIR traffic scene dataset. These videos are original and contain various scene conditions, such as complex illumination, night, curve lanes, lane changes, etc.

**Figure 11:**
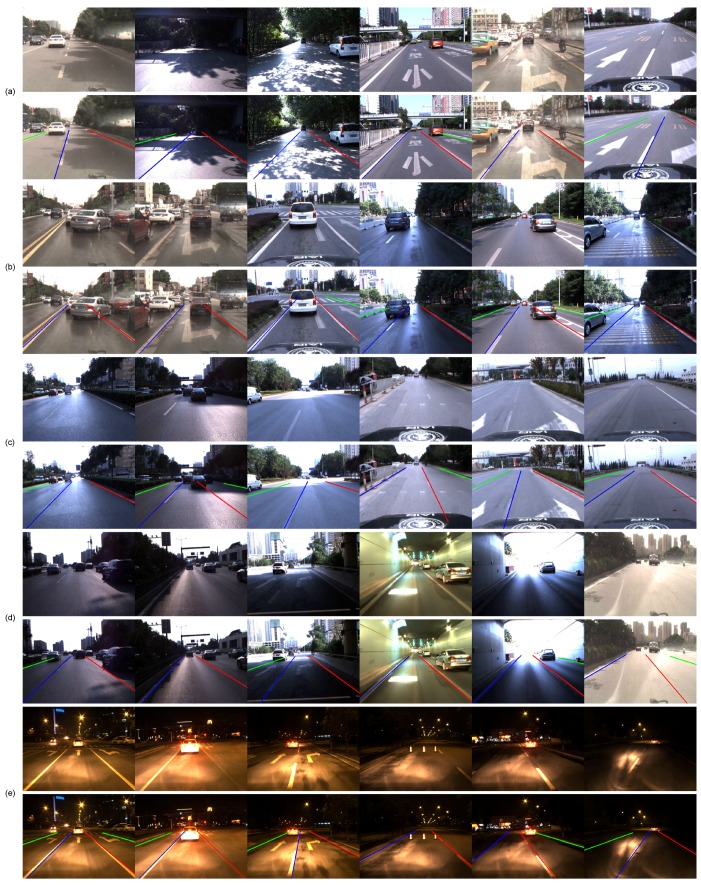
Examples of lane detection in various scenes. (**a**) Roads with shadows and paint; (**b**) Vehicle disturbance; (**c**) Worn lane boundaries; (**d**) Complex illumination; (**e**) Night.

**Figure 12:**
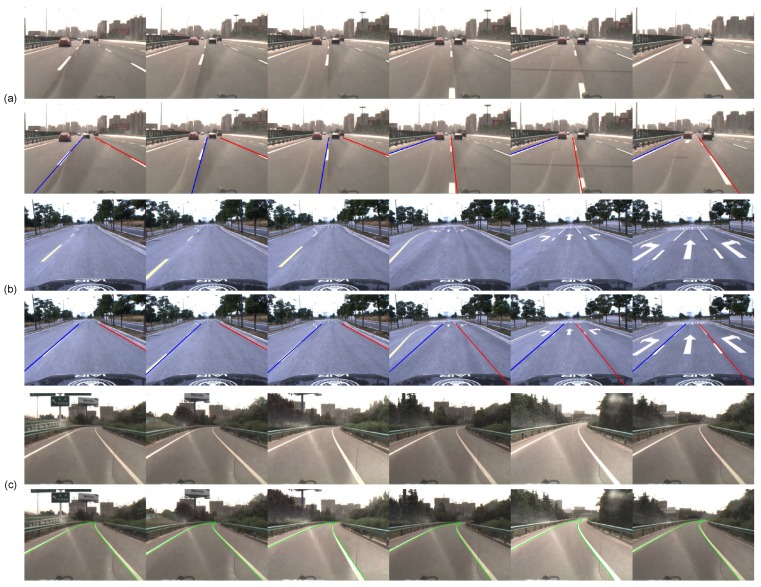
Examples of lane detection in road structure changes. (**a**) In the process of changing lanes; (**b**) Lanes suddenly become narrow; (**c**) Curved roads.

Table 2 presents the quantitative results of our method, and Figure 11 shows some examples of lane boundary detection in various scenes. Figure 12 shows some examples of lane boundary detection when the road structures change. Despite the various disturbances on highways, our method successfully detects almost all lane boundaries with a precision of 99.9%. Only 16 out of 14,182 are false alarms because a van was overtaking at a near distance. Even excessively ‘sufficient’ or weak visual cues exist in some challenging scenes, as illustrated in Figure 11; our method still exhibits excellent performance that benefits from STF. Our algorithm can also successfully detect lane boundaries with changing structures, as shown in Figure 12.

### 6.4. Experiment 2: Comparison with Other Methods

We compare our method to three other lane detection methods on the data from the XJTU-IAIR traffic scene dataset, namely He’s method [14], Bertozzi’s method [41] and Seo’s method [9]. He’s method [14] uses the Canny detector to extract edge features and Hough transformation to select lane boundaries. Bertozzi’s method [41] searches for the intensity bumps to detect lanes. Seo’s method [9] extracts lane features using a spatial filter and refines the feature by utilizing the driving direction.

**Figure 13:**
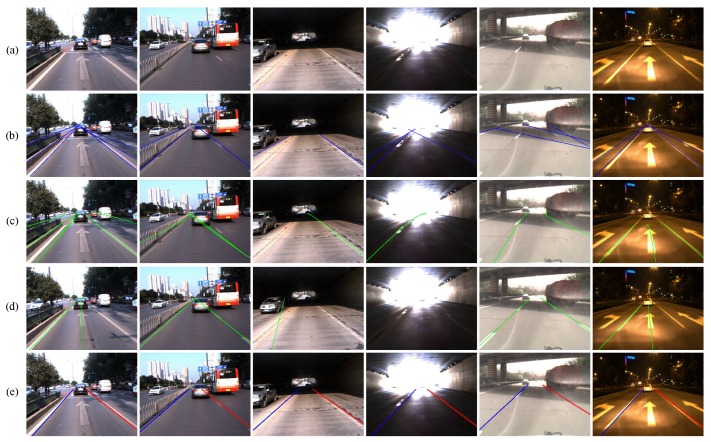
Examples of our method and other methods. (**a**) Original images; (**b**) He’s method [14]; (**c**) Bertozzi’s method [41]; (**d**) Seo’s method [9]; (**e**) Our method.

Table 3 presents the performance of each method in different traffic conditions, and Figure 13 shows some examples. Table 3 and Figure 13 show that our method is more robust than the other three methods in these challenging traffic conditions, which proves the strength of our knowledge-based filtering framework. For example, in the heavy urban video, which exhibits complex lane structures and visual cues, the performance of our method is much better than the other three methods. This is because our method combines the prior spatial-temporal knowledge to detect the lane boundaries rather than only utilizing the appearance features.

### 6.5. Experiment 3: Aly’s Dataset

We also test our method on Aly’s dataset [33]. The comparisons between our method and other methods are summarized in Table 4. For a fair comparison, we adopt the same evaluation criteria as in Aly’s method [33].

Aly’s dataset includes four videos. Our algorithm demonstrates good performance on Videos 1, 3 and 4. On Video 2, a large number of false positives exist because of crossroads with many cracks. The line segments at the cracks share the same direction and location with the real lane boundaries and, therefore, can hardly be filtered out by CPF and STF.

### 6.6. Experiment 4: Different Platforms

To transplant our algorithm to other computing platforms, we also test our method on Raspberry Pi 3 and ARK-10. Raspberry Pi 3 is a single board computer with a 1.2-GHZ CPU, and ARK-10 is an embedded industrial control computer with a 2.0-GHZ CPU.

In the test, we adopt two strategies for algorithm acceleration. Firstly, we resize the original image into 320×240. Secondly, on each resized frame, we set the region between the vanishing line and the bottom line as the searching region. This strategy prevents the algorithm from searching invalid areas where there is no lane boundary. Table 5 shows the performance and the speed of our algorithm on these two platforms. Our algorithm can achieve an FPS of about 18 on Raspberry Pi 3 and about 29 on ARK-10. Such a speed can basically meet the requirement of some real-time applications.

On the other hand, the low resolution of the video frames may cause some potential limitations. Some detailed information is not perceived on the low-resolution frames, which may lead to more false negatives in some situations with excessively weak visual cues.

## 7. Discussion and Conclusions

In this paper, we propose a spatial-temporal knowledge filtering method to detect lane boundaries in videos. The model unifies the feature-based detection and knowledge-guided filtering into one framework. Two filters are proposed to filter out the noisy line segments in the massive original line segment features. These two filters characterize the spatial structure constraint and temporal location constraint, which represent the prior spatial-temporal knowledge about lanes. The proposed method was tested on a large-scale traffic dataset, and the experimental results demonstrate the strength of the method. The proposed algorithm has been successfully applied to and tested on our autonomous experimental vehicle.

**Figure 14:**
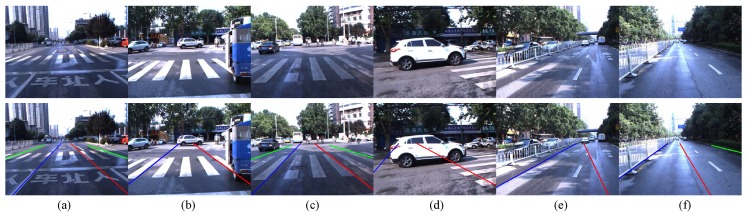
Examples of false positives. (**a**–**c**) Zebra crossings; (**d**,**e**) Crossroads; (**f**) Wet roads.

Our method may produce false results in some special traffic conditions, such as crossroads, zebra crossings and wet roads. Figure 14 shows some examples of false results. Our future work will focus on these issues and other intelligent vehicle-related topics, such as pedestrian action prediction, complex traffic scene understanding and 3D traffic scene reconstruction.

## Figures and Tables

**Table 1:** XJTU-IAIR traffic scene dataset.

Traffic Scenes	Highway	Urban	Illumination	Curve	Night	Changing Lanes
Frame Number	7091	96085	5245	418	7640	548

**Table 2:** The performance of our method in different scenes of XJTU-IAIR traffic scene dataset.

Traffic Scenes	Frames	GT	TP	FP	FN	Precision (%)	Recall (%)	FPS
Highway	7091	14,182	14,166	16	0	99.9	100	28
Moderate Urban	5579	10,157	9786	230	163	97.7	98.4	21
Heavy Urban	5880	10,616	9892	478	158	95.4	98.4	16
Illumination	1040	2080	2080	0	0	100.0	100.0	23
Night	804	1566	1566	10	0	99.4	100.0	35
Curve	418	836	822	4	10	99.5	98.8	25
Changing Lanes	548	1096	1096	0	0	100.0	100.0	28

**Table 3:** Comparison with other methods on different scenes of the XJTU-IAIR traffic scene dataset.

Traffic Scenes	Methods	Frames	GT	TP	FP	FN	Precision (%)	Recall (%)	FPS
Highway	Ours	999	1998	1998	0	0	100.0	100.0	30
	He’s [14]	999	1998	1805	183	172	90.8	91.3	14
	Bertozzi’s [41]	999	1998	1886	33	79	98.3	96.0	35
	Seo’s [9]	999	1998	1711	211	78	89.0	95.6	21
Moderate Urban	Ours	999	1998	1966	30	0	98.5	100.0	18
	He’s [14]	999	1998	1946	419	31	82.3	98.4	11
	Bertozzi’s [41]	999	1998	1649	236	152	87.5	91.6	34
	Seo’s [9]	999	1998	1363	124	511	91.7	72.7	14
Heavy Urban	Ours	899	1692	1621	89	26	94.8	98.4	19
	He’s [14]	899	1692	1609	608	69	72.6	95.9	11
	Bertozzi’s [41]	899	1692	1289	386	212	77.0	85.9	31
	Seo’s [9]	899	1692	970	295	441	76.7	68.7	15
Illumination	Ours	1040	2080	2080	0	0	100.0	100.0	23
	He’s [14]	1040	2080	1725	697	305	71.2	85.0	23
	Bertozzi’s [41]	1040	2080	1882	185	32	91.0	98.3	34
	Seo’s [9]	1040	2080	1506	448	128	77.1	92.2	17
Night	Ours	804	1566	1566	10	0	99.4	100.0	35
	He’s [14]	804	1566	1491	326	75	82.1	95.2	12
	Bertozzi’s [41]	804	1566	942	379	603	71.3	61.0	36
	Seo’s [9]	804	1566	1383	104	87	93.0	94.1	24
Changing Lanes	Ours	548	1096	1096	0	0	100.0	100.0	28
	He’s [14]	548	1096	876	368	81	70.4	91.5	13
	Bertozzi’s [41]	548	1096	1015	85	5	92.3	99.5	39
	Seo’s [9]	548	1096	975	99	24	90.8	97.6	21

**Table 4:** Comparison with other methods on Aly’s dataset.

Video	Methods	Frames	CR(%)	FPR(%)
1	Ours	250	99.8	1.9
	Aly’s [33]	250	97.2	3.0
	He’s [14]	250	87.6	25.9
	Seo’s [9]	250	87.6	10.8
2	Ours	406	92.6	6.8
	Aly’s [33]	406	96.2	38.4
	He’s [14]	406	82.4	54.8
	Seo’s [9]	406	89.1	9.2
3	Ours	336	98.2	1.3
	Aly’s [33]	336	96.7	4.7
	He’s [14]	336	74.0	27.7
	Seo’s [9]	336	81.8	5.3
4	Ours	232	97.8	2.2
	Aly’s [33]	232	95.1	2.2
	He’s [14]	232	85.1	25.2
	Seo’s [9]	232	88.8	5.2

**Table 5:** The performance of our method on different computing platforms.

Platforms	Frames	Resolution	Precision (%)	Recall (%)	FPS
Raspberry Pi 3	2587	320×240	99.0	100.0	18
ARK-10	2587	320×240	99.0	100.0	29
